# Cerebrospinal fluid dynamics at the lumbosacral level in patients with spinal stenosis

**DOI:** 10.1186/2045-8118-12-S1-P24

**Published:** 2015-09-18

**Authors:** Keewon Kim, Dong-Joo Kim, Kwang Dong Kim, Hack-Jin Lee, Chul-Ho Sohn, Sun-Gun Chung, Se-Woong Chun, Ho NamKoong

**Affiliations:** 1 Seoul National University Hospital, Korea, Republic of (South Korea; 2 Department of Brain and Cognitive Engineering, Korea University, Korea, Republic of (South Korea

## Introduction

The hydrodynamics of the cerebrospinal fluid (CSF) is well-known to contribute to neurological disorders of the brain. However, little attention has been paid to the CSF dynamics in the lumbosacral spine. Meanwhile, the pathomechanism of neurogenic claudication, a characteristic symptom of spinal stenosis, has not been clearly elucidated. In this study, we suspected that spinal stenosis may be associated with altered CSF dynamics and compared CSF flow velocities at the lumbosacral spinal level between patients with spinal stenosis and healthy controls, at rest and at claudication.

## Methods

Twelve subjects (4 patients with spinal stenosis and 8 healthy controls; 25-77 years old; 7 males) underwent phase-contrast magnetic resonance imaging (PC-MRI) to quantify CSF dynamics at the lumbosacral spinal level. Using PC-MRI, the CSF flow velocities were measured at the L2 and S1 levels. All of the subjects underwent PC-MRI at rest and after walking (to provoke neurogenic claudication in the patients).

## Results

The flow rate in the sacral spine (caudal peak flow: -0.25 ± 0.28 cm/s) was greatly attenuated compared to the flow in the lumbar spine (caudal peak flow: -0.93 ± 0.46 cm/s) in both patients and controls. The caudal peak flow was slower in patients (-0.65 ± 0.22 cm/s) than controls (-1.07 ± 0.49 cm/s). The difference between the L2 caudal peak flow became more pronounced after walking (-0.66 ± 0.37 cm/s in patients, -1.35 ± 0.52 cm/s in controls; p = 0.028). The sacral CSF flow after walking was barely detectable in patients (caudal peak flow: -0.09 ± 0.03 cm/s) compared with controls (caudal peak flow: -0.32 ± 0.26 cm/s). The severity of structural stenosis (area or AP diameter of the spinal canal) did not correlate with the flow velocities within the participants.

## Conclusions

CSF dynamics in the lumbosacral spine were more attenuated in patients with spinal stenosis than healthy controls in a manner that was not proportionate to the structural stenoses. After walking, the CSF flow rate did not exhibit an appropriate increase in patients experiencing claudication, whereas the flow rate did increase appropriately in controls. Altered CSF dynamics may partially explain the pathophysiology of spinal stenosis.
